# What can speech tell us about pain?

**DOI:** 10.1097/PR9.0000000000001293

**Published:** 2025-06-05

**Authors:** Adrian Gonzales, Kylie Yao, Adam Vogel, Natalia Egorova-Brumley

**Affiliations:** a Melbourne School of Psychological Sciences, University of Melbourne, Melbourne, Australia; b Department of Audiology and Speech Pathology, University of Melbourne, Melbourne, Australia; c Redenlab Inc., Melbourne, Australia

**Keywords:** Acute pain, Speech, Pain sensitivity

## Abstract

**Introduction:**

Pain experiences are subjective and can vary significantly between individuals, yet pain is universally communicated using speech. Analysis of speech itself may be clinically informative about pain. Speaking itself may also relieve pain as it does during basic vocalisations. Whether complex speech relieves pain is not known. Understanding speech when pain is being experienced and whether certain speech features are associated with general pain sensitivity holds promise for identifying pain markers.

**Objectives:**

We investigated the (1) effect of acute pain on speech characteristics, (2) effect of speech on pain intensity, and (3) association between pain-free speech characteristics and pain sensitivity.

**Methods:**

Speech metrics were derived from 25 healthy adults who performed speech tasks that fit along a continuum of cognitive complexity, with and without acute exposure to noxious heat stimuli. Pain ratings were also recorded.

**Results:**

Speech was pain-sensitive: under acute pain syllabic rate and speech-to-pause ratio increased during a monologue task, while mean sound pressure level decreased during vowel production. Exploratory correlations between pain-free speech characteristics and baseline pain sensitivity were significant. Increased speech-to-pause ratio was associated with increased cold pain sensitivity, while decreased mean sound pressure level was related to higher pressure pain sensitivity and impaired conditioned pain modulation. Producing a monologue reduced pain more than saying /a:/.

**Conclusion:**

Speech can serve as a marker of acute pain. It also correlates with pain sensitivity, suggesting that speech could be useful for pain assessment and management.

## Abstract 2

Natural speech characteristics can be used as biomarkers of pain and are linked to baseline pain sensitivity, and speech production reliably reduces pain intensity.

## 1. Introduction

Pain comprises myriad physiological, cognitive, and social factors.^18^ Its multidimensional nature underscores the challenges associated with accurate pain assessment and treatment in the clinic, which often involves subjective verbal self-reports by those in pain.^13,56,62,70^

Cognitive and neuromuscular processes—which are impaired in various clinical conditions—are inextricably linked to measurable acoustic features in speech.^24,59^ Acoustic analysis of speech has shown promise in neurology,^6,17,37,38,42,52,53,63,67^ speech pathology,^9,23,25,29,30^ and psychopathology.^14,15,34^ Encouragingly, recent speech analysis techniques have demonstrated high accuracy in using normative and pathological speech assays to distinguish between neurological conditions.^53^ This is a promising frontier but is hitherto underexplored in the context of pain assessment.^5^ Moreover, the use of acoustic analysis of speech holds promise as a novel measure in clinical pain assessment and as a complement to more subjective self-reports.

### 1.1. Changes in speech during pain

Both acute pain and chronic pain can alter speech characteristics,^16^ suggesting inherent links between speech and pain are worthy of investigation. Rowbotham et al. (2014) found acute pressure pain to be correlated with increased speech production (word count) and articulation difficulty. Conversely, slowed speech is observed in individuals with chronic pain.^47^ Other reported speech–pain links include increased fundamental frequency (pitch),^41^ altered Mel-frequency cepstral coefficients (representation of vocal tract dynamics),^10,60^ and other prosodic changes.^19,39^

Data on the pain–speech link are mostly reported in the context of nonexperimental research.^5,10,39,43,61^ Previous research has examined speech metrics in diverse populations, including individuals with chronic pain, children, and adults, reflecting the heterogeneity of pain conditions^35^ and vocalisations.^16,19^ Although this diversity provides valuable context, it can introduce variability that complicates the identification of salient patterns of speech.^51^ By focusing on healthy adults, this study aims to establish foundational speech metrics in the absence of confounding factors, offering a baseline that can enhance the interpretation and applicability of findings in more complex populations.

### 1.2. Effect of speech on pain

Pain vocalisations have been studied in the context of eliciting helping behaviour from others.^40,49,54^ However, pain can also be modulated via top–down cognitive processes,^68^ so speech—a cognitively demanding task—can be used for pain management. Even simple vocalisations (eg, “ow”,^58^ swearing^44^) can reduce pain intensity. Systematic contrasts between basic vocalisations and complex forms of speech, in terms of pain reduction, are lacking.

### 1.3. Speech and pain sensitivity

Links between heightened pain sensitivity and chronic pain are observed whereby individuals with chronic pain exhibit higher pain sensitivity.^1,4,31,33,55^ In clinical practice, a holistic approach is often adopted to assess pain risk.^20,22,48,69^ Yet, by understanding how pain sensitivity is reflected in speech, it may be possible to identify individuals predisposed to pain, yielding a more proactive approach to pain assessment.

This study had 3 aims to investigate in healthy adults: (1) the effect of acute pain on speech metrics, (2) compare the analgesic effects of simple vs complex speech on acute pain, and (3) to explore links between speech metrics and baseline pain sensitivity measures.

## 2. Methods

### 2.1. Standard protocol approvals, registrations, and patient consents

Ethical approval was granted by the University of Melbourne Human Research and Ethics Committee (HREC). The study adhered to the Declaration of Helsinki (1964). Healthy adults (N = 25) were recruited via online advertisements targeting university students. Participants provided written informed consent and were reimbursed for the one-hour study. A screening survey included questions about long-term medical and psychological issues, current medications, chronic pain and peripheral neuropathy, as well as the Pittsburgh Sleep Quality Inventory to identify individuals sleeping <6 hours. No participants were subsequently excluded.

### 2.2. Equipment

Speech was recorded using the Redenlab software (Desktop) (Redenlab Inc, Melbourne, Australia) via an AKG 520C condenser microphone (Harman, Los Angeles, CA) and Roland Rubix 24 USB Audio Interface (Roland Corporation, Osaka, Japan). Speech was sampled at 44.1 KHz and 16-bit quantization.

Participants' pain sensitivity was measured using heat and cold stimuli administered through the Medoc Pathway Pain & Sensory Evaluation System (Medoc, 2005). A heat probe of approximately 1 × 1 inch was placed on the skin of each participant's forearm of the nondominant hand. Ischemic pain sensitivity was measured using the AlgoMed FPIX Computerised Pressure Algometer (Medoc, 2010), involving the application of a 1 × 1-cm pressure algometer to the palm of the nondominant hand.

### 2.3. Speech assessment

Participants completed a battery of 5 speech tasks of varied cognitive-motor complexity.^66^ Speech tasks included a 60-second monologue about a chosen topic; reading a written passage (“The North Wind and the Sun”) (139 orthographic syllables) aloud for 60 seconds; sustaining a vowel (say /a:/) for 5 seconds; saying the days of the week (Monday–Sunday) (lasting approximately 5 seconds); and a diadochokinetic task, repeating syllables (“pa-ta-ka…”) as quickly and clearly as possible for 10 seconds. These tasks are known to be stable in the absence of true change, with known variability in short and intermediate retest intervals.^65^

### 2.4. Baseline pain sensitivity assessment

Measures of pain sensitivity (thresholds) involved the application of 3 stimulus types: heat, cold, and physical pressure. Stimulus intensity ramped up (for heat and pressure) or down (for cold) in a linear fashion from a baseline of 32°C or 0 kilopascals (kPa) for pressure, until participants indicated that they felt pain. Pain sensitivity was measured 3 times to derive an average pain threshold for each stimulus modality.

Temporal summation measures intrinsic pain regulation through repeated noxious stimuli. Participants experienced tonic heat (eg, 45°C), calibrated to their static pain sensitivity. Pain intensity was rated from 1 to 10 (10 = “extreme pain”) every 30 seconds over 120 seconds. An increase in pain ratings indicated higher pain facilitation.^11^ The difference between the first and last ratings was used for analysis.

Conditioned pain modulation (CPM) measures pain regulatory function. Conditioned pain modulation is represented by reduced acute pain intensity at 1 location (eg, pressure pain applied to left arm) in response to a different pain stimulus at a distal location (eg, heat pain applied to right arm).^36^ In the current paradigm, we measured pressure pain before and after the application of 120 seconds of noxious heat (*M* = 44.96, *SD* = 2.49). The difference between the second and the first pressure threshold represented the magnitude of CPM (pain regulation).

### 2.5. Experimental pain

For the experiment, the temperature (°C) of noxious heat stimuli per participant was calibrated according to individual thresholds for moderate pain. Participants experienced up to seven 10-second rounds of noxious heat, with heat intensity increasing by 1°C each round. The baseline temperature (first round of heat administration) was set to each participant's initial heat pain threshold. During each round, participants verbally rated their felt pain on a numerical scale out of 10, where 0 = no pain and 10 = extreme pain. This process concluded once a subjective threshold for moderate pain—a “6 out of 10”—was obtained. This temperature was used for all pain stimuli in the pain-only and speech and pain conditions during the experiment. The duration of heat stimulation was matched to the duration of the specific speech tasks (eg, 5 seconds for the vowel task, 60 seconds for the monologue task).

### 2.6. Procedure

Both speech and pain data were collected at the Melbourne School of Psychological Sciences, Australia. Speech data were processed by Redenlab Inc and The University of Melbourne using automated acoustic pipelines.

Participants completed 5 speaking tasks with and without exposure to noxious heat stimuli. Stimulus intensity was calibrated to each participant's threshold for moderate pain (*M* = 44.96°C, *SD* = 2.49). Speech was recorded with and without pain (ie, pain and speech vs speech-only condition), as well as pain being experienced with and without speech (ie, pain and speech vs pain-only condition).

**Figure 1.**
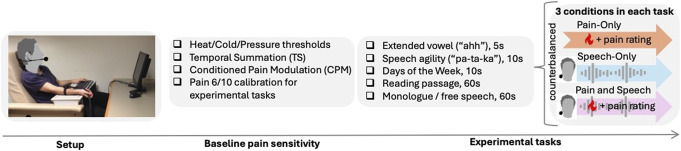
Timeline of the study protocol. The heat probe was placed on the volar forearm of the nondominant hand. Participants followed instructions presented on a desktop computer and spoke into a microphone. First, participants' baseline pain sensitivity was measured and the temperature for heat stimuli to be used for the experimental pain was individually calibrated (stimulus eliciting a pain rating of 6 out of 10 was selected). After this, 5 experimental tasks were completed. Each task involved 3 conditions: pain-only, speech-only, and pain and speech, with the order counterbalanced. The duration (in seconds) of noxious heat administration matched the duration of the speech task. For example, in the monologue task, participants would be asked (1) to speak on the topic of their choice for 60 seconds (speech-only), (2) experience heat pain for 60 seconds and rate their pain at the end of the session (pain-only), and (3) speak on the topic of their choice while experiencing heat pain for 60 seconds rating their pain at the end of the session (pain and speech). Every participant underwent all 3 conditions for each of the 5 experimental tasks.

The monologue task (60 seconds) and the reading task (60 seconds) were recorded once per condition (speech-only and pain and speech). To mitigate order and practice effects in analysis, the shorter speech tasks (10-second speech agility task, 5-second vowel/days of the week tasks) were repeated twice per condition and the second instances for each condition were used for the analysis of speech metrics. The same number of pain administrations in the pain-only condition for each of the 5 speech tasks was provided to facilitate planned comparisons between conditions. The duration of acute pain matched the duration of the speech task (Fig. 1).

In small untimed breaks between trials, participants verbally rated the intensity of the pain after exposure to noxious heat stimuli in the pain and speech and pain-only conditions, using the Gracely Pain Intensity Scale.^13^

### 2.7. Speech analysis

Seven speech metrics considered most likely to be affected by pain^10,19,39,40,41,46,47,60^ were selected for analysis. These included speech rate—(1) the *number of spoken syllables per second* and (2) the *speech-to-pauses ratio*; and 5 acoustic features—(3) the *coefficient of variance in fundamental frequency (f0 cov)*, variability of the frequency at which the vocal folds vibrate when voiced speech sounds are made (Hz), proxy of vocal control relating to pitch, (4) *cepstral peak prominence*, or measure of the degree of harmony within a voice sample, obtained using a Fourier transform of the logarithm power spectrum (dB), *a proxy of voice quality*, (5) *mean harmonics-to-noise ratio (HNR), a proxy of voice quality*, or the ratio of the acoustic energy of the harmonic components to that of the noise (dB), (6) *mean sound pressure level (SPL)*, a proxy of vocal control relating to loudness, and (7) *mean Mel frequency cepstral coefficient (MFCC2)*, or the second component making up the representation of the short-term power spectrum of a sound, a proxy of vocal tract dynamics. All 7 metrics were available for the monologue and reading tasks. For the days and agility tasks, only speech rate metrics were used, and for the vowel task, only voice quality features were used.

For the correlational analysis between speech and baseline pain sensitivity, analysis was focused on the metrics derived from the monologue and the extended vowel tasks, as these demonstrated significant differences between the speech-only and pain and speech conditions. We focused on the speech-only condition as it represents normal speech (unaltered by acute pain) to correlate baseline speech with baseline pain sensitivity.

### 2.8. Statistical analysis

Statistical analyses on the pain–speech relationship were conducted using R statistical software (R Core Team, 2021) and SPSS (version 29; IBM Corp., New York, 2023), with accepted levels of significance at *P* < 0.05.

The sample, N = 25, was determined sufficient for this study and was based on similar studies involving experimentally induced acute pain to measure the effects of pain on speech (with *d* = 0.6, alpha level = 0.05, and a desired power = 0.8, the minimum sample required is N = 23).^46^ Paired *t*-tests were conducted to evaluate the effect of pain on speech metrics comparing “speech-only” and “pain and speech” conditions for all speech tasks.

A repeated-measures ANOVA was conducted to determine the speech task associated with the largest amount of pain reduction (ie, the effect of each of the 5 speaking tasks on reported pain intensity in the “pain-only” vs “pain and speech” conditions). Paired *t*-tests were used to compare mean pain ratings between the “pain-only” vs “pain and speech” conditions. Since basic vocalisations were previously shown to induce analgesia,^58^ the magnitude of pain reduction from the basic extended vowel task (/a:/) was compared with the magnitude of pain reduction in the other speech tasks using paired *t*-tests.

Correlation analyses were conducted to explore the relationship between speech metrics in the monologue and vowel tasks with pain sensitivity measures (heat pain threshold, cold pain threshold, pressure pain threshold, temporal summation, and conditioned pain modulation). A sensitivity power analysis (G*Power 3.1) suggested that with N = 25 achieving 80% power with alpha = 0.05 for a 2-sided test would allow us to detect effects r < −0.396 and r > 0.396 (lower and upper critical r).

## 3. Results

### 3.1. Participants

Pain and speech data were acquired from healthy adult participants (N = 25) age 19 to 50 years (*M* = 26.61, *SD* = 7.32), including 16 females (*M* = 25.21, *SD* = 4.58) and 9 males (*M* = 28.78, *SD* = 10.22).

### 3.2. The effect of pain on speech metrics

**Figure 2.**
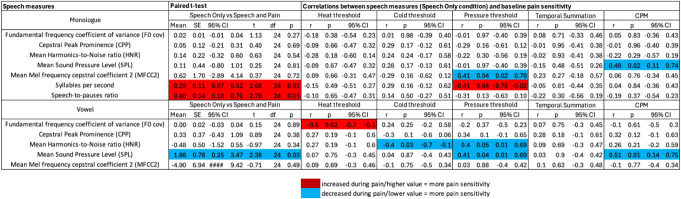
Speech features affected by the presentation of acute pain (showing differences in the speech-only vs speech and pain conditions) or associated with baseline pain sensitivity measures of heat pain, cold pain, pressure pain, temporal summation, and conditioned pain modulation (CPM) in the monologue and vowel tasks. Significant effects (*P* < 0.05, 2-tailed) are highlighted in red and blue. Confidence intervals (CI) are shown for all measures.

Significant differences were observed comparing the speech-only vs pain and speech conditions in the monologue and the vowel tasks only, with no significant differences in the reading, agility, or days of the week tasks. Speech rate was significantly higher in the pain and speech condition, as measured by increased number of syllables per second [*t*(1,24) = −2.676, *P* = 0.013, 95% CI [0.07, 0.52], *d* = 0.535] and an increased speech-to-pauses ratio [*t*(1,24) = −2.780, *P* = 0.010, 95% CI [0.1, 0.7], *d* = 0.556] in the monologue task. Mean SPL decreased during pain and speech in the vowel task [*t*(1,24) = 2.382, *P* = 0.025, 95% CI [0.25, 3.47], *d* = 0.476] (Fig. 2).

### 3.3. The effect of speech task on reported pain intensity

Mean pain ratings were significantly lower in the pain and speech condition relative to the pain-only condition, for each speaking task, including the extended vowel (*M* = 1.62; *t*(49) = 4.33, *P* < 0.0001, 95% CI [0.87, 2.37], *d* = −0.17); agility task (*M* = 1.7; t(49) = 6.29, *P* < 0.0001, 95% CI [1.16, 2.24], *d* = −0.26); days of the week (*M* = 2.09; *t*(49) = 5.60, *P* < 0.0001, 95% CI [1.34, 2.84], *d* = −0.46); reading (*M* = 2.48; *t*(24) = 6.19, *P* < 0.0001, 95% CI [1.65, 3.31], *d* = −1.24), and monologue (*M* = 3.00; *t*(24) = 5.84, *P* < 0.0001, 95% CI [1.94, 4.06], *d* = −1.17).

**Figure 3.**
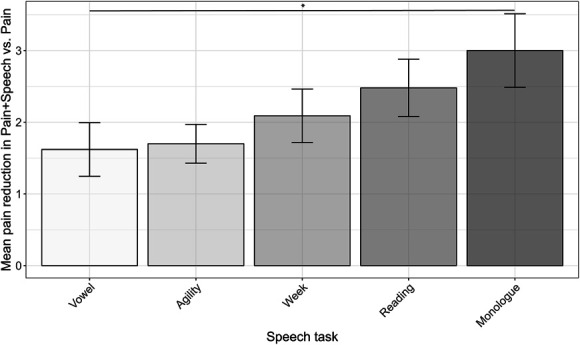
Magnitude of heat pain reduction for each speech task. Planned analysis involved the comparison of heat pain reduction in the vowel task to all other tasks. For N = 25, producing a monologue elicited greater pain reduction compared with the vowel task (*P* < 0.043). Error bars are represented as standard error (SEM), and asterisk (*P < 0.05) indicates significant differences.

A repeated-measures ANOVA did not reveal any significant main effect of the type of speech task on self-reported pain intensity (*P* = 0.05). The monologue task reduced pain significantly more than saying /a:/ (*M* = 1.38; *t*(24) = 2.14, *P* < 0.043, 95% CI [0.05, 2.71], *d* = −0.43) (Figure 3). Eleven participants (44% of the sample) showed a 4-point difference in pain reduction (*M* = 4.05, *SD* = 1.63) in favour of the monologue vs the vowel task. All other tasks did not indicate significant differences with the vowel task in terms of reported pain (*P* > 0.05).

### 3.4. The relationship between baseline pain sensitivity and speech metrics

Significant correlations between pain sensitivity measures and speech metrics derived from the speech-only condition were observed in both the monologue and the vowel tasks, as shown in Figure 2. Significant vowel task speech correlations were found with the heat pain threshold: *f*0 cov [r(25) = −0.455, *P* = 0.022]; cold pain threshold: mean HNR [*r*(25) = −0.444, *P* = 0.026]; pressure threshold: mean HNR [*r*(25) = 0.4, *P* = 0.048] and mean SPL [*r*(25) = 0.406, *P* = 0.044]; and conditioned pain modulation: mean SPL [*r*(25) = 0.509, *P* = 0.009]. Monologue task speech correlations were found with the pressure threshold: mean MFCC2 [*r*(25) = 0.413, *P* = 0.04 and syllables per second [*r*(25) = −0.413, *P* = 0.04]; and conditioned pain modulation: mean SPL [*r*(25) = 0.481 *P* = 0.015]. The sensitivity power analysis suggested that we were only able to detect effects r = ±0.396, and all the reported results indeed show greater effect sizes. We did not have the power to observe smaller effects. It is therefore important to underscore that the current results are exploratory only.

## 4. Discussion

The relationship between experimentally induced pain and speech characteristics in healthy adults was analysed using a comprehensive battery of speech tasks. The study investigated the effects of acute pain on speech characteristics, the effect of basic vs complex speech in pain reduction, and links between speech and measures of pain sensitivity. The vowel and monologue tasks provided the most consistent associations between speech features, acute pain, and higher pain sensitivity, namely, there was a decreased mean SPL (in both the vowel and monologue tasks) and increased speech rate (syllables per second in the monologue), suggesting these metrics are linked to pain. Pain was consistently reduced when experienced in conjunction with producing speech, indicating basic and complex speech reliably reduce pain intensity, with the monologue reducing pain more than saying /a:/. This suggests that tasks requiring complex cognitive and motor planning and execution are superior in reducing pain compared with less cognitively loaded speech tasks.

### 4.1. Changes in speech during pain

A previous study observed slowed speech in individuals with chronic pain,^47^ attributing speech patterns to a combination of centrally mediated motor inhibition, impaired selective attention, and/or physical bracing, all being features of chronic pain. However, it remained unclear, whether slower speech was a feature of pain experience or attributable to age (ie, mean age: 48.5, upper range: 81) or cognitive decline (ie, chronic pain has been associated with a higher cognitive decline, particularly in processing speed).^45^

We tested pain in healthy young participants, demonstrating higher speech rate during pain. Faster speech in the monologue also aligned with other studies that observed increased word count, cospeech gesturing, and articulation difficulty while describing intense acute pressure pain.^46^ The previous study was conducted using acute experimental pain with healthy young adults (mean age: 19 years), although there were noteworthy differences between study designs. Speech tasks were time-limited and thus constrained total word production in this study, indicating that speech rate—a function of motor output—is altered by pain. Participants were also required to perform speech while physically still (no cospeech gesturing) and without conversing with a human interlocutor (ie, de-emphasising the sociocommunicative aspects of speech) during pain. Similar speech characteristics were altered despite differences in pain modalities (ie, heat vs pressure pain) and the valence of speech content (ie, a neutral monologue vs describing pain). These distinctions support that pain influences speech through automatic neurophysiological responses and sociocognitive processes that drive pain communication. Results are also consistent with observations that cognitive-motor processes in complex speech are more likely to be influenced by externally induced perturbations, relative to automatic speech, which is free of meaning or phonetic complexity.^66^

Previous studies observed trends in altered mean HNR and *f*0.^19^ This study did not observe such effects, although they were relevant to pain sensitivity as discussed below. However, SPL (speech volume) was lowered during the vowel task, suggesting it is possible that acute pain perturbs motor processes involved in the inspiration and expiration of air during extended sound production.

### 4.2. Changes in pain during speech

Pain intensity was consistently reduced when participants engaged in speech compared with experiencing pain in isolation. Basic vocalisations (/a:/) provided analgesic effects, aligning with observations that swearing^44^ saying “ow”^58^ reduces acute pain intensity. In aggregate, these observations indicate that all forms of vocal expression (basic and complex) can mitigate pain.

Speech tasks requiring novel language formulation alongside coordination of complex motor sequences, as in a monologue, provide greater pain reduction than less cognitively loaded tasks such as sustained phonation (saying /a:/). Pain reduction was significantly greater for monologues than vowels: an average difference of 1.38 points (noting that overall monologue task-related average pain reduction was by 3 out of 20 points), with 44% of participants reporting a categorical shift in pain intensity, eg, “strong” to “moderate”, or “mild” to “weak” pain.

Differences in complex and simple speech for pain reduction can be understood through motor planning, working memory, and attention. Tasks demanding more working memory and attention elicit greater analgesia during acute pain.^8,28^ Monologues require verbal fluency, memory recall, and coherent integration,^2,3^ while also diverting attention to nonpainful stimuli^27,64^ (eg, retelling weekend events). This suggests that the monologue's cognitive and motor complexity, unrelated to pain, offers greater analgesia compared with vocalising /a:/, which is more synonymous with pain.

Individuals with chronic pain show heightened attention to somatosensory stimuli.^7^ However, the relationship between somatosensory awareness and pain is complex. Somatosensory awareness may influence pain sensitivity, potentially mediated by psychological factors such as pain catastrophising.^12,32,50^ Mindfulness-based interventions demonstrate how enhanced somatosensory awareness supports pain interpretation and emotional regulation, fostering adaptive coping strategies.^26^ This highlights the need for further research into the role of somatosensory awareness in experimental pain contexts.

However, no significant differences in pain reduction were detected when comparing the vowel task with other more complex speech forms. Understanding the contribution of speech towards pain reduction could open new avenues for accessible and effective pain management strategies, especially where pain is unavoidable (eg, clinical settings).

### 4.3. Speech and pain sensitivity

There were moderately strong correlations between speech metrics derived from the speech-only condition and baseline pain sensitivity measures. Faster speech (syllables per second) in the monologue task and lower SPL (softer speech) in the vowel task were associated with higher pain sensitivity. The directions of these effects were consistent with the effects of pain on speech in the speech and pain condition. In addition, a lower SPL in the monologue and vowel tasks was associated with lower conditioned pain modulation (CPM), which is characteristic of chronic pain.^57^ However, there was no observable link between speech and pain facilitation (temporal summation).

Higher pain sensitivity correlated with lower HNR (voice quality), lower MFCC2 (vocal tract variability), and higher *f*0 cov (pitch variation). Lower HNR was linked to greater pressure and cold pain sensitivity, aligning with findings in chronic and ischemic pain.^19^ Harmonics-to-noise ratio is also understood as central among speech characteristics in voice alterations during stress.^21^ Higher pitch variability (*f*0 cov) may reflect centrally mediated pain sensitivity, despite no observed direct effects of pain on *f*0 cov. Lower MFCC2 during the monologue was associated with higher pressure pain sensitivity, suggesting monotonous speech may be linked to pain susceptibility as in other studies,^10^ noting that there was no directly observed effect of acute pain on MFCC2.

Speech metrics showed stronger associations with pressure pain sensitivity and CPM (measured as the difference between pressure after heat stimuli). This suggests speech metrics are particularly sensitive to pressure pain, aligning with studies showing increased speech production under acute ischemic pressure.^46^ Speech relies on complex neuromuscular coordination, which may underlie its sensitivity to pressure pain. Future research should explore whether ischemic pain uniquely affects speech compared with other pain modalities.

These observations are highly encouraging of the value of speech acoustic analysis in yielding markers of pain sensitivity, which could prove useful in evaluating pain susceptibility before the development or worsening of pain.

### 4.4. Limitations and future research

The modest sample size (N = 25) limited the ability to confirm moderating effects of speech complexity on pain perception (*P* = 0.05). Despite this, consistent reductions in pain across speech tasks suggest speech reliably reduces acute pain. Correlations between baseline speech features and pain sensitivity should be considered exploratory only. Although underpowered for directional analysis, the findings align with the known pain–speech link and propose measures for future investigation.

The study focused on acute pain in healthy adults, limiting generalisation to chronic pain. Considering contrasting evidence from previous studies,^47^ which found that slowed speech correlated with greater chronic pain intensity, there is a need for systematic comparisons between different pain types and populations. Future studies could use experimental designs that juxtapose normative and clinical groups to identify distinct speech metrics associated with chronic pain.

## 5. Conclusion

This study undertook acoustic analysis of a comprehensive speech battery to explore whether speech is altered by pain and affects pain. We demonstrated that (1) alterations in certain speech metrics provide signatures of a painful experience, (2) producing complex speech reliably reduces acute pain intensity, and (3) certain speech characteristics could be linked to pain sensitivity.

By exploring speech markers of pain and pain sensitivity, this study lays the groundwork for further exploration into the interplay between speech characteristics and pain experiences.

This study provides a method to deepen understanding of patient pain beyond traditional self-reports. Accurate assessment is crucial for tailoring pharmacological and nonpharmacological treatments. The findings hold potential for clinical applications, improving pain assessment, treatment strategies, and patient outcomes. Further research and implementation could transform pain management for chronic and acute conditions.

## Disclosures

The authors declare that there are no competing interests associated with this work.
